# Application of multi-edge HERFD-XAS to assess the uranium valence electronic structure in potassium uranate (KUO_3_)

**DOI:** 10.1107/S1600577521012431

**Published:** 2022-01-01

**Authors:** René Bes, Gregory Leinders, Kristina Kvashnina

**Affiliations:** a Department of Physics, University of Helsinki, PO Box 64, FI-00014 Helsinki, Finland; b Helsinki Institute of Physics, PO Box 64, FI-00014 Helsinki, Finland; c Belgian Nuclear Research Centre (SCK CEN), Institute for Nuclear Materials Science, B-2400 Mol, Belgium; d The Rossendorf Beamline at ESRF, The European Synchrotron, CS40220, 38043 Grenoble Cedex 9, France; e Institute of Resource Ecology, Helmholtz Zentrum Dresden-Rossendorf (HZDR), PO Box 510119, 01314 Dresden, Germany; f Department of Chemistry, Lomonosov Moscow State University, Moscow 119991, Russia

**Keywords:** X-ray absorption spectroscopy, HERFD-XANES, uranium, electronic structure, *FDMNES*

## Abstract

The uranium valence electronic structure in the prototypical undistorted perovskite KUO_3_ is reported on the basis of a comprehensive experimental study using multi-edge HERFD-XAS and relativistic quantum chemistry calculations based on density functional theory. Very good agreement is obtained between theory and experiments, including the confirmation of previously reported Laporte forbidden *f*–*f* transitions and X-ray photoelectron spectroscopic measurements. Many spectral features are clearly identified in the probed U-*f*, U-*p* and U-*d* states and the contribution of the O-*p* states in those features could be assessed. The octahedral crystal field strength, 10*Dq*, was found to be 6.6 (1.5) eV and 6.9 (4) eV from experiment and calculations, respectively. Calculated electron binding energies down to U-4*f* states are also reported.

## Abstract 2

By combining HERFD-XAS at the uranium *L*_1_-, *L*_3_- and *M*_4_-edge with relativistic quantum chemistry calculations the uranium valence electronic structure in KUO_3_ could be assessed in detail. The contribution of uranium and oxygen states in observed spectral features is discussed.

## 1. Introduction

Uranium compounds are usually associated with nuclear applications, such as nuclear fission for energy generation, but for many decades they also triggered interest from more fundamental aspects. In particular, uranium can induce very versatile physico-chemical properties due to the wide range of possible oxidation states offered by its [Rn]7*s* ^2^6*d* ^1^5*f* ^3^ electronic ground state configuration. Additionally, uranium 5*f* electrons show an apparent duality in localization, being often found in radially dispersed and hybridized bands in the vicinity of the Fermi level, whereas sometimes they remain localized (Guziewicz *et al.*, 2004 ▸; Teterin *et al.*, 1981 ▸; Teterin & Teterin, 2004 ▸). One consequence is the ability of the 5*f* electrons to participate in the conduction band and to remain localized simultaneously, producing rather complex and still unclear behaviour of the uranium bonding nature with a mixed covalent/ionic character (Kaltsoyannis, 2013 ▸). Despite the large amount of crystallographic, physical, chemical and thermodynamic data available today (Grenthe *et al.*, 2006 ▸), understanding of the uranium electronic structure is far from complete. Experimental studies typically require dedicated laboratories, owing to the natural radioactivity of uranium, and, in addition, theoretical calculations are revealed to be extremely complicated because of comparable magnitudes of the crystal field, spin–orbit coupling and the electron–electron repulsion interactions. The influence of the latter effect can be significantly reduced by exploring pure pentavalent U(V) compounds. Indeed, the U(V) valence state corresponds to a simpler electronic configuration [Rn]5*f* ^1^, where no electron–electron repulsion interactions are expected, at least within the 5*f* shells.

Uranium occurs in numerous ores and minerals, as well as in seawater, but most often it is present in a hexavalent U(VI) state, or in a mixture of tetra-, penta- or hexavalent states (Grenthe *et al.*, 2006 ▸). Single-valence uranium compounds are much less widespread, the most common being uranium(IV) oxide (UO_2_) known for its application as nuclear fuel in light-water reactors. The pentavalent U(V) state has been identified in oxidation products of UO_2_, such as U_4_O_9_, U_3_O_7_ and U_3_O_8_, but here always occurs as a mixture with tetra- or hexavalent uranium (Kvashnina *et al.*, 2014 ▸; Leinders *et al.*, 2017 ▸, 2021 ▸). One exception might be the, somewhat obscure, U_2_O_5_ phase, which has been reported to exist under very specific conditions (Hoekstra *et al.*, 1970 ▸; Gouder *et al.*, 2018 ▸). More commonly, stable pentavalent uranium compounds occur in ternary systems of uranium and oxygen with one additional cation (*e.g.* from the alkali metal group, or certain alkaline earth and transition metals, as well as some of the rare-earth elements) (Selbin & Ortego, 1969 ▸). However, oxygen non-stoichiometry, leading to a mixed-valence character, may sometimes develop in these ternary compounds (Grenthe *et al.*, 2006 ▸). Potassium uranate, KUO_3_, which crystallizes in a prototypical cubic perovskite structure, contains a pure U(V) ion as confirmed by recent X-ray absorption spectroscopy studies at the uranium *L*_3_-edge (Soldatov *et al.*, 2007 ▸; Leinders *et al.*, 2020 ▸) and *M*_4_-edge (Leinders *et al.*, 2017 ▸), and from U-4*f* X-ray photoelectron spectroscopy studies (Lopez *et al.*, 2017 ▸; Liu *et al.*, 2009 ▸). Other metal uranate systems, such as NaUO_3_, RbUO_3_ and TlUO_3_, similarly are pentavalent uranium compounds, and have been investigated in multiple studies (Aravamudan *et al.*, 1978 ▸; Chippindale *et al.*, 1989 ▸; Soldatov *et al.*, 2007 ▸; Misra *et al.*, 2008 ▸; Sanyal *et al.*, 2017 ▸; Butorin *et al.*, 2016 ▸). However, these compounds exhibit more distorted crystal structures with local atomic disorder, owing to the values of the ionic radii which deviate from the optimal ratio in perovskite systems (Kassan-Ogly & Naish, 1986 ▸). KUO_3_, on the other hand, is an example of a prototypical cubic perovskite with no local disorder, which makes it an ideal U(V) reference material.

The magnetic properties of KUO_3_ were extensively studied using neutron diffraction, magnetic susceptibility measurements as a function of temperature, as well as electron paramagnetic resonance (Dickens & Powell, 1991 ▸; Hinatsu, 1994 ▸; Hinatsu *et al.*, 1998 ▸; Van den Berghe *et al.*, 2004 ▸). A sharp spike in the magnetic susceptibility at *ca*. 17 K was found and attributed to an antiferromagnetic-type magnetic ordering at low temperature (Hinatsu, 1994 ▸). Van den Berghe *et al.* (2004 ▸) later experimentally confirmed such magnetic ordering to be G-type antiferromagnetic with an orthorhombic magnetic unit cell, in line with recent theoretical results by Dorbane *et al.* (2019 ▸). However, some uncertainty remains on the effective magnetic moment carried by the U(V) ion, which has been reported (from experiment) to be equal to 0.20(3)μ_B_ (Van den Berghe *et al.*, 2004 ▸) and 0.66μ_B_ (Hinatsu, 1994 ▸). In addition, theoretical calculated values vary between 0.9932μ_B_ and 1.2500μ_B_ depending on the type of method used (Dorbane *et al.*, 2019 ▸).

Concerning the electronic structure of KUO_3_, only few studies are reported in the literature. Hinatsu revealed forbidden-Laporte *f*–*f* transitions in optical absorption spectra originally reported by Kemmler-Sack (1968 ▸), which were interpreted on the basis of an octahedral crystal field model. Through this model, Kemmler-Sack deduced the crystal field parameters θ and Δ, and the spin–orbit coupling constant ξ, which were found to be equal to 0.58062 eV, 0.41349 eV and 0.23507 eV, respectively (Hinatsu, 1994 ▸). Electronic calculations based on density functional theory (DFT) indicate that the valence band top and the conduction band bottom are mainly dominated by U-*f* states with a very small contribution of the O-*p*, U-*p* and U-*d* states (Azam & Reshak, 2014 ▸; Dorbane *et al.*, 2019 ▸). These calculations agree on the fact that KUO_3_ is a semiconductor with an indirect band gap. However, the calculated band gap is reported as 4.652 eV (Azam & Reshak, 2014 ▸) and varies from 0.5077 eV to 5.1354 eV depending on specific conditions of the used DFT method (Dorbane *et al.*, 2019 ▸). Covalent bonding between U and O atoms because of orbital hybridization is predicted in all calculations. Finally, anisotropic hyperfine interactions with predominant π character were observed by ^17^O nuclear magnetic resonance studies of ^17^O-enriched KUO_3_ (Eastman *et al.*, 1971 ▸). The major contribution to the hyperfine interaction was found to involve excited uranium orbitals such as 7*s* or 6*d* states and/or exchange polarization of core electrons.

The lack of consensus on some of the fundamental properties of the alkali metal uranate KUO_3_ demonstrates the need to investigate further the electronic and magnetic properties of this interesting compound. By combining uranium *L*_1_-, *L*_3_- and *M*_4_-edge experiments, one probes 7*p*, 6*d* and 5*f* states, respectively, giving an almost complete overview of uranium valence electronic structure, excluding 7*s* states. We are reporting here results of our study on the uranium valence electronic structure in KUO_3_ by means of multi-edge high energy resolution fluorescence detected X-ray absorption spectroscopy (HERFD-XAS) and state-of-the-art relativistic quantum chemistry calculations based on DFT.

## 2. Materials and methods

### 2.1. Sample preparation

Polycrystalline KUO_3_ powder was prepared by mixing stoichiometric amounts of U_3_O_8_ and K_2_CO_3_ powders, and performing heat treatments in a Carbolite TZF1800 tube furnace. Firstly, a portion of depleted UO_2+*x*_ powder (nuclear-grade impurity) (Leinders *et al.*, 2015 ▸), supplied by FBFC International (Belgium), was oxidized to U_3_O_8_ by treating at 500°C for 4 h in a dynamic air atmosphere (N_2_/21 vol% O_2_). Secondly, stoichiometric amounts of U_3_O_8_ and K_2_CO_3_ (ACS Reagent, ≥99.0%), obtained from Sigma-Aldrich (Belgium), were intimately mixed using a zirconia mortar and pestle, and the mixture was annealed at 800°C for 10 h. A reducing atmosphere (−400 kJ mol^−1^ at 800°C) was applied by flushing the furnace with a gas mixture of Ar/0.5 vol% O_2_ (519 ml min^−1^) and Ar/5 vol% H_2_ (481 ml min^−1^). The used gases were of high purity (99.9992%) and flow rates were accurately controlled using Bronkhorst EL-FLOW mass flow controllers.

Phase purity of the sample was confirmed from X-ray powder diffraction (PANalytical X’Pert Pro), see Figure 8 in the supporting information.

Sample preparation for X-ray absorption spectroscopy at the ESRF consisted of mixing the KUO_3_ powder (30–50 mg) with boron nitride powder (98%), purchased from Sigma-Aldrich (Belgium). The sample holder used at beamline ID26 required to compact the resulting powder into a thin pellet. The sample holder used at beamline BM20 consisted of a polypropylene disc with a small recess in which the powder was directly inserted. Both sample holders were sealed with Kapton foil.

### 2.2. High energy resolution fluorescence detected X-ray absorption spectroscopy

Uranium *L*_1_- and *L*_3_-edge XANES measurements were performed at BM20 (The Rossendorf Beamline) (Matz *et al.*, 1999 ▸; Scheinost *et al.*, 2021 ▸) of the European Synchrotron Radiation Facility (ESRF) operating at an electron beam energy of 6 GeV, in Grenoble, France. The incident energy was scanned using a Si(111) monochromator. HERFD-XANES spectra were collected at room temperature using an X-ray emission spectrometer equipped with five Si(220) crystal analyzers with 0.5 m bending radius, and a silicon drift X-ray detector in a vertical Rowland geometry (Kvashnina & Scheinost, 2016 ▸). For uranium *L*_3_-edge HERFD-XANES measurement, the spectrometer was tuned to the maximum of the U *L*α_1_ (2*p*_3/2_–3*d*_5/2_ transition at 13.614 keV) X-ray emission line using the 〈880〉 reflection at a Bragg angle of 72°. For the uranium *L*_1_-edge HERFD-XANES measurement, the spectrometer was tuned to the maximum of the U *L*β_4_ (2*s*_1/2_–3*p*_1/2_ transition at 16.575 keV) X-ray emission line using the 〈10 10 0〉 reflection at a Bragg angle of 77°. The detected intensity was normalized to the incident flux. Beam size was estimated to be 200 µm (vertically) by 450 µm (horizontally). The beamline settings available at the time of the measurements did not allow ultra-high-energy resolution in HERFD mode to be achieved. Nevertheless, the total experimental energy broadening (incident energy convoluted with emitted energy and core-hole lifetime broadening) of 3.9 eV and 12.5 eV were still below the core-hole lifetime broadening of the U *L*_3_-edge (∼8.2 eV) and *L*_1_-edge (∼17.5 eV). The resolution can be further improved for example at the U *L*_3_-edge by using a Si(311) crystal monochromator, a Ge(777) crystal analyzer with 1 m bending radius, and by reducing the beam size below 100 µm. Energy calibration was achieved through the Y *K*-edge excitation energy (17.038 keV) and through the Ru *K*-edge excitation energy (22.117 keV) of metallic yttrium and ruthenium foils placed in the beam path for the uranium *L*_3_- and *L*_1_-edge, respectively.

The U *M*_4_-edge HERFD-XANES measurements were performed at the ID26 beamline (Gauthier *et al.*, 1999 ▸) of the ESRF. The U *M*_4_-edge (3.725 keV) incident energy was selected using the Si(111) double-crystal monochromator. Rejection of higher harmonics was achieved by three silicon mirrors at 3.5 mrad working under total reflection. Beam size was estimated to be 200 µm (vertically) by 500 µm (horizontally). HERFD-XANES spectra were measured at room temperature using an X-ray emission spectrometer equipped with five 1 m bending radius Si(220) crystal analyzers and a silicon drift X-ray detector in a vertical Rowland geometry. The spectrometer was tuned to the maximum of the U *M*β (3*d*_3/2_–4*f*_5/2_ transition, at 3.3374 keV) X-ray emission line using the 〈220〉 reflection (analyzer crystals at a Bragg angle of 75.4°). The detected intensity was normalized to the incident flux. The total experimental energy broadening (incident energy convoluted with emitted energy and core-hole lifetime broadening) was evaluated at 0.7 eV.

It has to be mentioned that, following the allowed electronic dipolar transition, the main differences between HERFD-XANES experiments at the uranium *L*_1_-edge, *L*_3_-edge and *M*_4_-edge lie in the fact that different density of states are being probed, namely uranium 7*p*, 6*d* and 5*f* states, respectively. Those states are likely to show individual sensitivity towards the uranium oxidation state as a function of their involvement in bonding to ligands, the presence of crystal-field effects and the core-hole broadening affecting the overall resolution of useful features.

### 2.3. Electronic structure calculations

**Figure 1:**
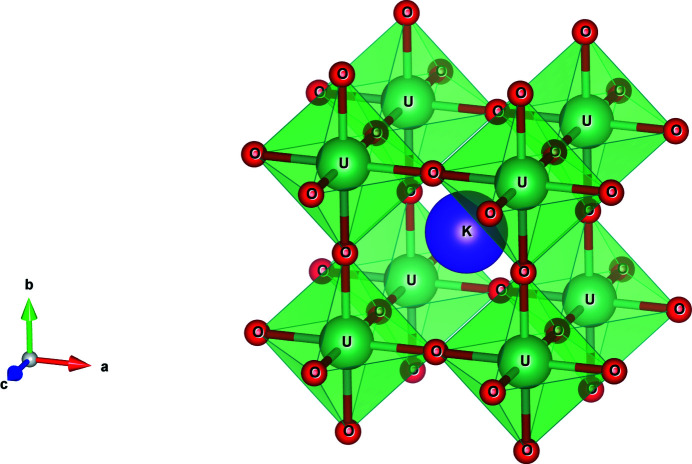
Representation of the KUO_3_ unit cell, which exhibits an ideal, cubic perovskite structure. The oxygen atoms form a framework of corner-linked oxygen octahedra around each U atom. The K atom is positioned in the central cuboctahedral void formed by the vertices of the anion octahedra. Figure created using the *VESTA3* software (Momma & Izumi, 2011 ▸).

The XANES theoretical calculations were performed using the *Finite Difference Method for Near-Edge Structure* (*FDMNES*) code (Bunău & Joly, 2009 ▸). KUO_3_ is an alkali-metal uranate crystallizing in a prototypical undistorted perovskite-type structure ( 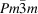 ), in which U atoms are octahedrally coordinated by oxygen (see Fig. 1 ▸) situated at 2.37 Å at room temperature. The K atoms are cuboctahedrally coordinated (12-fold) by oxygen atoms, at a distance equal to 3.04 Å.

An atomic cluster of 9 Å was used in self-consistent-field calculations using the Dirac–Slater approach. The Poisson equation was solved to obtain the Coulomb potential from the superposed self-consistent atomic densities in the considered cluster. The energy-dependent exchange-correlation potential was evaluated using the local density approximation, and constructed using both the real Hedin–Lundquist and Von Barth formulations. These calculations were based on static atom supercells of hundreds of atoms, and thermally induced disorder was not considered. Because of the presence of heavy nuclei (U), spin–orbit effects were taken into account but no spin-polarization effect has been noticed from the comparison of spin-polarized spectra. Finally, calculations were performed with and without the quadrupolar transition probability in order to assess the 6*d* and 5*f* contribution on the final spectra at the U *L*_1_ and *L*_3_-edges, respectively.

## 3. Results and discussion

### 3.1. Uranium *L*_1_-edge HERFD-XANES

**Figure 2:**
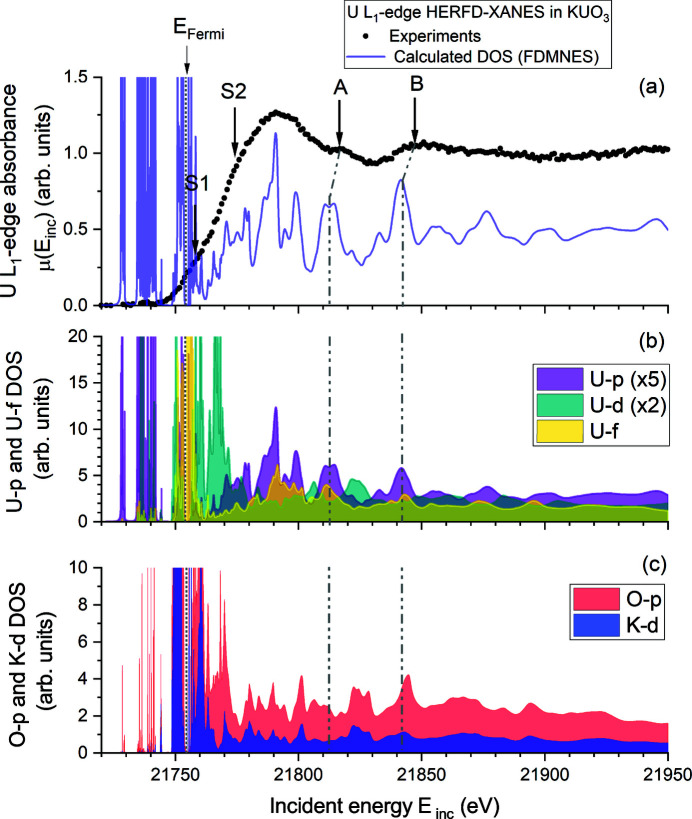
(*a*) Uranium *L*_1_-edge HERFD-XANES and the corresponding simulated spectrum using *FDMNES*. (*b*, *c*) Calculated U-*p*, U-*f*, O-*p* and K-*d* partial density of states, to allow direct comparison with the experimental XANES spectrum. The Fermi energy level is indicated with a vertical dotted line. Vertical dashed-dotted lines indicating the position of features A and B in partial DOS are also present to guide the eyes. The intensity of U-*p* and U-*d* partial DOS in subplot (*b*) were here multiplied by 5 and 2, respectively, to better distinguish each contribution. We refer to Figure 6 of the supporting information for the other calculated partial DOS.

Experiments at the U *L*_1_-edge (∼21758 eV) probe the U 7*p* states following the allowed electronic dipolar transition 2*s* → (*n*)*p*. Conventional XANES characterization is not often performed at this uranium edge due to the very large core-hole lifetime broadening of about 17 eV, which hinders resolving useful features. However, by applying HERFD-XANES, several features can become visible thanks to the tremendous gain in energy resolution by the virtual reduction of core-hole broadening. The obtained HERFD-XANES spectrum of KUO_3_ is shown in Figure 2 ▸ together with the simulated spectrum and the calculated partial density of states (DOS) of selected orbitals. The maximum of the white line occurs at 21791 (1) eV, while the maximum of the first derivative, which corresponds to the uranium *L*_1_-edge energy position *E*_0_, is found at 21770 (1) eV.

Two post-edge features, indicated as A and B on Figure 2 ▸, are resolved and their energy position can be identified at 21817 (1) eV and 21850 (1) eV, respectively. Good agreement is observed between calculated and experimental spectra in terms of number of features and their relative intensity. Some discrepancy can be understood due to the fact that the *FDMNES* calculations used here are based on DFT which is by definition a fundamental state theory at 0 K, and therefore it is not necessarily adapted to XAS which is probing an excited state, here at room temperature. The post-edge features A and B are not calculated exactly at their corresponding energy position observed in the experiment, but occur around 21812.8 (2) eV and 21841.9 (2) eV, respectively. The calculated DOS (see Figure 2 ▸, and additionally Figure 7 of the supporting information) indicate that these features are essentially caused by U-*p*, with a potential smaller contribution of the U-*f* states. Moreover, in feature B the O-*p* and K-*d* states are overlapping with the aforementioned U states in energy scale, which demonstrates their role in uranium bonding with oxygen and potassium neighbours.

In addition to the two post-edge features, two shoulders marked as S1 and S2 in Figure 2 ▸ on the lower energy side of the white line, at 21757 (1) eV and 21770 (1) eV, respectively, are also observed. The former shoulder S1 would typically be attributed to a pre-edge feature, *i.e.* a possible quadrupolar transition 2*s* → (*n*)*d*, in line with U-*d* DOS. However, calculations without quadrupolar transition are not demonstrating its U-*d* states origin. Assigning it to an octupolar transition 2*s* → (*n*)*f* can also be ruled out, given its significant strength and its occurrence in dipole-only calculations. The most plausible interpretation remains a U-*p* origin given the presence of such states in partial U-*p* DOS (see Figure 7 of the supporting information). Nevertheless, the relative U-*p* DOS intensities are not sufficient to solely explain the observed intensity of S1. Usually not well reproduced by our calculations, it is likely that such a feature is enhanced by the mixing of U-*d*/*f* and U-*p* states with potential effect of hybridization to the O-*p* states, as revealed by the overlapping U-*p*, U-*f* and O-*p* DOS in this energy range. Further studies at the U *L*_1_-edge are therefore required to confirm such hypothesis. The latter shoulder S2 at 21770 (1) eV seems also related to the splitting of U-*p* states into two main bands, one at around 21774 (3) eV and the other at 21790 (3) eV. Both bands contribute significantly to the overall shape of the white line and link to the energy position of the white line maximum and *L*_1_-edge energy position *E*_0_ (*i.e.* the first derivative).

### 3.2. Uranium *L*_3_-edge HERFD-XANES

**Figure 3:**
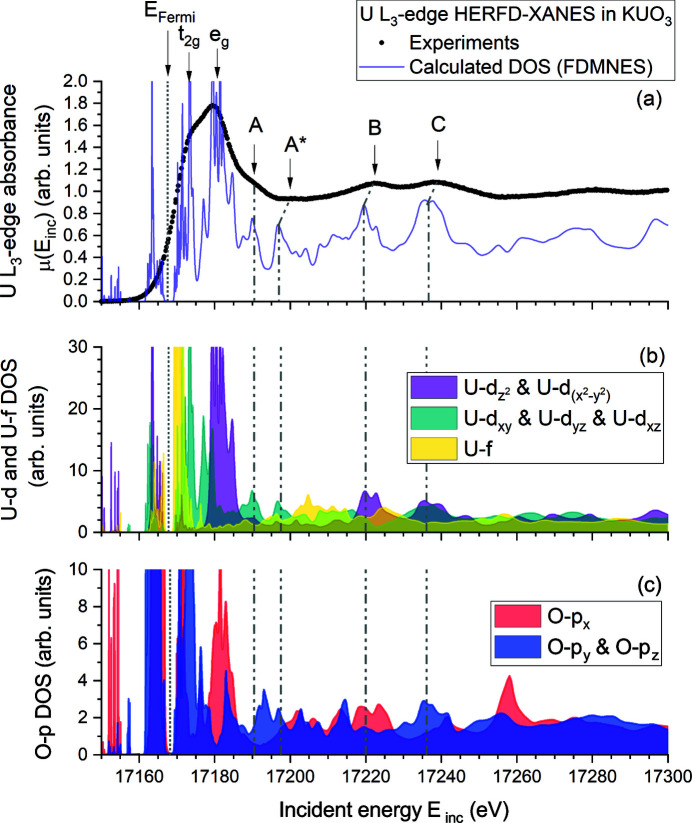
(*a*) Uranium *L*_3_-edge HERFD-XANES and the corresponding simulated spectrum using *FDMNES*. (*b*, *c*) Calculated U-*d*, U-*f* and O-*p* partial DOS, here expressed as cubic harmonics (except U-*f*), to allow direct comparison with the experimental XANES spectrum. The Fermi energy level is indicated with a vertical dotted line. Vertical dashed-dotted lines indicating the position of features A, A 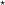 , B and C in partial DOS are also present to guide the eyes. We refer to Figure 6 of the supporting information for the other calculated partial DOS.

Experiments at the U *L*_3_-edge (∼17166 eV) probe the U 6*d* states following the allowed electronic dipolar transition 2*p* → (*n*)*d*. The uranium *L*_3_-edge is often used for XANES and EXAFS characterizations thanks to its limited core-hole lifetime broadening of about 8 eV, which is significantly lower than at the U *L*_1_-edge. Again, by using HERFD-XANES instrumentation, more features can be resolved thanks to the tremendous gain in energy resolution by the virtual reduction of core-hole broadening. The obtained HERFD-XANES spectrum of KUO_3_ is shown in Figure 3 ▸ together with the simulated spectrum and the calculated partial DOS of selected orbitals. Very good agreement between experiments and calculation is demonstrated. The maximum of the white line occurs at 17179.6 (5) eV, while the maximum of the first derivative, which corresponds to the uranium *L*_3_-edge energy position *E*_0_, is found at 17169.9 (5) eV.

In the post-edge region of the experimental spectrum (see Figure 3 ▸), up to four features can be identified, here designated A, A 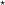 , B and C. In general, all observed spectral features are reproduced by the calculations in terms of number and relative intensity. A comparison between the experimentally observed and calculated energy position of the post-edge features is reported in Table 1 ▸. Some discrepancy remains on the respective positions, especially for A 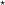 , but with differences of the order ∼1 eV the agreement is better than at the U *L*_1_-edge.

To gain more insight on the nature of the spectral features, a distinction between cubic harmonics of U-*d* states and O-*p* states was made in Figure 3 ▸. Features A and A 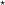 can be considered to originate mainly from U-*d* states, with no significant contribution from O-*p* states. Feature B is essentially related to U- 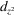 and U- 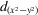 , with also a significant contribution of O-*p*_*x*_. Feature C appears to result from a mixture of all U-*d* electrons, together with contributions of O-*p*_*y*_ and O-*p*_*z*_. Therefore, features B and C are most relevant with respect to uranium–oxygen bonding.

In addition to those post-edge features, the improved energy resolution in HERFD-XANES experiments allows to distinguish a bimodal white line shape. The occurrence of a quadrupolar transition 2*p* → (*n*)*f* as previously reported on UO_2_, [UO_2_py_5_][KI_2_py_2_] and UO_2_(NO_3_)_2_(H_2_O)_6_ by Vitova *et al.* (2010 ▸), and later also on U_4_O_9_ and U_3_O_8_ (Kvashnina *et al.*, 2014 ▸), and [Ni(H_2_O)_4_]_3_[U(OH,H_2_O)(UO_2_)_8_O_12_(OH)_3_] (Bès *et al.*, 2016 ▸), is not well resolved, despite the resolution gain in our experiments. Indeed, there is a partial overlap between U-5*f* and U-6*d* electrons as a consequence of the 6*d* electrons strong crystal-field splitting expected when considering the undistorted oxygen octahedral geometry around uranium atoms in the crystal structure of KUO_3_ (Kemmler-Sack, 1968 ▸). As a result, the U- 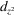 and U- 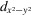 orbitals, the so-called *t*_2*g*_, are pulled to lower energy, partially overlapping (and probably hybridizing) with U-*f* orbitals. At the same time, U-*d*_*xy*_, U-*d*_*yz*_ and U-*d*_*yz*_ orbitals, the so-called *e*_*g*_, are pushed to higher energy. The crystal field strength, 10*Dq*, can be deduced from the energy separation between *t*_2*g*_ and *e*_*g*_ (see Table 1 ▸), where comparable values of 6.6 (1.5) eV and 6.9 (4) eV are found from experiment and calculations, respectively.

### 3.3. Uranium *M*_4_-edge HERFD-XANES

**Figure 4:**
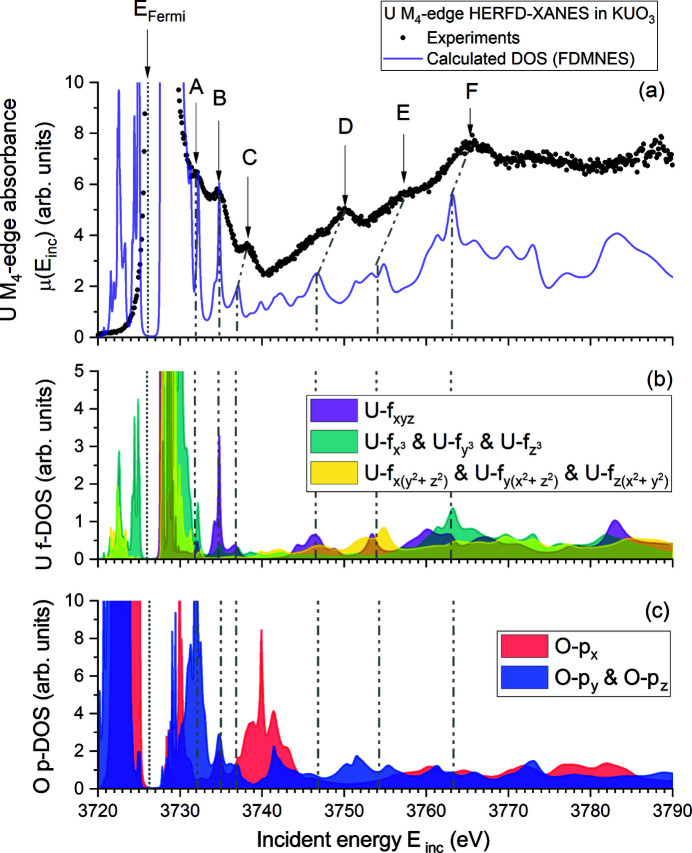
(*a*) Uranium *M*_4_-edge HERFD-XANES and the corresponding *FDMNES* calculated spectrum (non convoluted). The absorbance scale was limited in this plot to better distinguish the numerous satellite features (denoted as A, B, C, D, E and F). We refer to Figure 7 in the supporting information for the full scale absorption spectrum. (*b*, *c*) Calculated U-*f* and O-*p* partial DOS, here expressed as cubic harmonics, to allow direct comparison with the experimental XANES spectrum. The Fermi energy level is indicated with a vertical dotted line. Vertical dashed-dotted lines indicating the position of features A, B, C, D, E and F in partial DOS are also present to guide the eyes. We refer to Figure 6 of the supporting information for the other calculated partial DOS.

Experiments at the U *M*_4_-edge (∼3725 eV) probe the U 5*f* states following the allowed electronic dipolar transition 3*d* → (*n*)*f*. This technique, and especially the application of HERFD-XANES, has gained much attention over the past decade as more and more facilities offered its use on radioactive materials. By probing directly the U 5*f* states and having a formidable spectral resolution down to 1 eV and lower (Kvashnina *et al.*, 2014 ▸), it presents great advantages over the U *L*-edges with respect to chemical state determination in uranium compounds (Leinders *et al.*, 2017 ▸, 2020 ▸). The obtained HERFD-XANES spectrum of KUO_3_ is shown in Figure 4 ▸ together with the simulated spectrum and partial DOS of selected orbitals. Very good agreement between experiments and calculation is also demonstrated. The maximum of the white line occurs at 3726.7 (1) eV, while the maximum of the first derivative, which corresponds to the uranium *M*_4_-edge energy position *E*_0_, is found at 3726.4 (1) eV.

Thanks to the tremendous gain in energy resolution, several features (here assigned A to F) are clearly visible beyond the white line in Figure 4 ▸. These post-edge features are well reproduced by the calculations, but features C to F remain slightly off in energy position, with differences of the order 1–3 eV as reported also in Table 2 ▸. The good agreement between calculations and experiment allows to confidently assign spectral features to the U-*f* DOS, which are further divided as a function of the cubic harmonics. Feature A can be attributed to U- 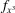 , U- 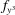 and U- 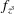 states, with significant hybridization to O-*p*_*y*_ and O-*p*_*z*_ states. Features B and C are composed of U-*f*_*xyz*_ states, with additional contribution from all O-*p* states, but feature C includes also significant contribution of U- 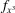 , U- 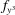 and U- 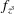 states. It is furthermore expected that the crystal field splitting is playing a significant role in the distribution and cubic harmonic nature of features A, B and C (Butorin, 2020 ▸). The features D and E relate to mixing of U-*f*_*xyz*_, U- 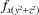 , U- 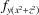 and U- 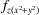 states, with additionally a significant contribution of O-*p*_*y*_ and O-*p*_*z*_ states which appears more pronounced in feature E. Finally, feature F is essentially composed of U- 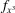 , U- 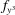 and U- 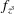 states with contribution of all O-*p* states. In conclusion, the observed hybridization between O-*p* and U-*f* states in the U *M*_4_-edge HERFD-XANES post-edge spectral features demonstrates a strong relation to the U–O bonding nature. Consequently, one may expect their occurrence, intensity and position to be sensitive to changes in U–O bonding and ligand geometry around uranium sites.

**Figure 5:**
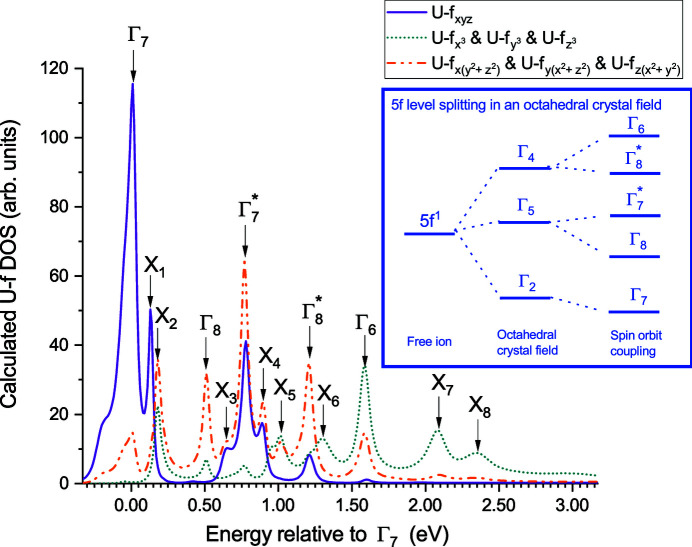
Uranium 5*f* level crystal field splitting as deduced from the calculated DOS. Those peaks are contributing to the white line intensity in the U *M*_4_-edge HERFD-XANES spectra. U-*f* states are here represented as cubic harmonic.

The experimentally observed U *M*_4_-edge white line displays an asymmetric sharp peak at 3726.7 (1) eV with a full width at half-maximum of about 1.5 eV [see Figure 7 in the supporting information, and Leinders *et al.* (2017 ▸)]. Its slight broadening is essentially due to a small shoulder clearly visible on its high energy side which is probably the consequence of the uranium 5*f* level crystal field splitting in addition to spin–orbit coupling. Such crystal field splitting is expected to be strong when considering the 5*f* ^1^ electronic configuration of uranium in an octahedral ligand geometry. Unfortunately, despite the resolution gain in HERFD-XANES, the core-hole lifetime broadening still prevents distinguishing the degeneracy breaking within the 5*f* bands resulting from this crystal field. Therefore, only insights obtained through calculations are available here. The calculated U-*f* DOS contributing to the white line is shown in Figure 5 ▸ alongside a schematic recalling the 5*f* ^1^ octahedral crystal field and spin–orbit coupling splitting.

Several isolated peaks are clearly visible in the calculated DOS, demonstrating the strength of the degeneracy breaking of 5*f* bands in KUO_3_. Their energy relative to the ground states, *i.e.* the first peak Γ_7_, are reported in Table 3 ▸. The five main peaks, denoted as Γ_7_, Γ_8_, 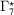 , 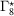 and Γ_6_, can be attributed to the five theoretically expected Γ_*n*_ states. A comparison of their energy position with the experimentally observed forbidden Laporte *f*–*f* transition Γ_7_ → Γ_*n*_ reported by Kemmler-Sack (1968 ▸) and by Kanellakopulos *et al.* (1980 ▸) supports well this assumption. Moreover, Γ_7_ and 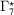 both display a U-*f*_*xyz*_ character, with additional contribution of U- 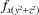 , U- 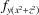 and U- 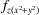 states, especially for 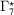 . States Γ_8_ and 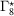 have a U- 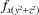 , U- 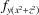 and U- 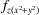 character with minor contribution of other U-*f* states, while Γ_6_ has a more pronounced U- 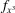 , U- 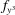 and U- 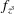 character with a small contribution of U- 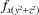 , U- 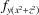 and U- 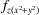 states. Therefore, the U-*f* character of these peaks, as deduced from our DOS calculations, further support the split state identification.

In addition to the five main Γ_*n*_ peaks, eight minor peaks, denoted *X*_*n*_ with *n* ranging from 1 to 8, are also predicted by our calculations. Almost all of them can be attributed to further degeneracy breaking of the five main peaks. *X*_1_ is related to the ground state Γ_7_ with major contribution of U-*f*_*xyz*_ states. *X*_2_ occurs very close to Γ_7_, but has no U-*f*_*xyz*_ states character. Instead, it resembles the character of Γ_8_. According to Allen *et al.* (1981 ▸), Γ_8_ and 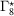 are likely to split if the octahedral coordination is distorted. However, no experimental evidence for distortion of the octahedron in KUO_3_ has been reported at room temperature (Chippindale *et al.*, 1989 ▸; Dickens & Powell, 1991 ▸; Soldatov *et al.*, 2007 ▸). Therefore, no distortion of the oxygen octahedra was taken into account in our calculations. *X*_3_ is rather weak in our calculations but its energy position corresponds well to a level not seen by Kemmler-Sack (1968 ▸) but reported by Kanellakopulos *et al.* (1980 ▸). Kanellakopulos *et al.* attributed *X*_3_ to further splitting of Γ_8_, but our calculations would predict a 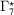 character due to the strong contribution of U-*f*_*xyz*_ states. Theoretical calculations by Hinatsu *et al.* (1998 ▸) predict inversion of Γ_8_ and 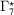 when the relative magnitudes of the crystal field with octahedral symmetry and spin–orbit coupling interactions decreased. Such an inversion occurs when the spin–orbit coupling constant is very low compared with the crystal field strength, and can be ruled out here when considering the observed energy of the *f*–*f* transition Γ_7_ → Γ_*n*_. *X*_4_ is very similar to *X*_3_, and can be assigned to 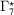 . *X*_5_ is a mixture of all U-*f* states except U-*f*_*xyz*_, similar to Γ_6_, but being situated between 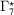 and 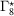 . Its attribution to any main peak splitting is ambiguous. *X*_6_, as well as *X*_7_ and *X*_8_, show only a U- 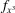 , U- 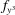 and U- 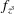 character, which also makes their association with any of the main peak unclear. Moreover, *X*_7_ and *X*_8_ are situated in the UV–Vis–NIR region where strong absorption has been reported in KUO_3_ by Kemmler-Sack (1968 ▸), Kanellakopulos *et al.* (1980 ▸) and Allen *et al.* (1981 ▸). Such absorption is not only attributed to charge transfer transitions, but also to Laporte-allowed uranium 5*f* → 6*d* transitions. Those transition are likely to be very intense, especially because *X*_7_ and *X*_8_ occur in regions where 5*f* and 6*d* hybridization are observed in our DOS calculations.

### 3.4. Electronic structure of filled orbitals

Given the good agreement between our calculations and experimental data above the Fermi level, one can also consider comparing the electronic structure of the filled orbital to additional experimental data, such as electron binding energies obtained using X-ray photoelectron spectroscopy (XPS). To the best of our knowledge, only Liu *et al.* (2009 ▸) and Lopez *et al.* (2017 ▸) have reported XPS data for KUO_3_. In both studies the U 4*f* doublet was measured using the Al *K*_α_ emission line (*KL*_2, 3_) as excitation source. Their measured electron binding energies and the ones deduced from our calculations are reported in Table 4 ▸.

Clearly, there is a lack in XPS experimental data for most of the electron binding energies. The XPS results from Liu *et al.* and Lopez *et al.* are in good agreement, especially when considering the energy differences between the U-4*f*_5/2_ and U-4*f*_7/2_ lines. However, the calculated binding energies are shifted by a few eV when comparing with experimental data. This may be expected when considering that the energy scale origin is different in our calculations and in the XPS measurements. Indeed, the binding energies in Liu *et al.* (2009 ▸) and Lopez *et al.* (2017 ▸) are given relatively to the carbon 1*s* peak position taken at 285.0 eV for energy calibration, while our calculated values are relative to the Fermi level. Moreover, our calculations are not taking into account the core-hole created during the absorption process, which may be not fully screened in both XAS and XPS experiment. The core-hole can lead to significant shifts in electron binding energies as well as satellite peaks in XPS spectra. By taking into account the core-hole within our XAS calculations, the U-4*f* electron binding energies would be increased by about 27 eV, which shows their high sensitivity to screening effects. However, the agreement between XAS experiments and calculations with such a core-hole is worse, in particular at the U *M*_4_-edge.

## 4. Conclusions

A comprehensive analysis of multi-edge high energy resolution fluorescence detected X-ray absorption data obtained at the uranium *L*_1_-edge, *L*_3_-edge and *M*_4_-edge for KUO_3_ is reported. The focus has been to evaluate the electronic structure of uranium in KUO_3_ by comparing theoretical calculations with experimental data. Despite the relative simplicity of our DFT calculations, very good agreement was found. At the uranium *L*_1_-edge, a pre-edge feature was observed as a shoulder on the white line due to core-hole broadening effects, but its origin is still unclear. Two U-*p* character post-edge features were identified, with a different contribution of O-*p* states to each of them. At the uranium *L*_3_-edge, the strength of the crystal field splitting of U-6*d* states is evaluated at 6.6 (1.5) and 6.9 (4) eV from experiment and calculations, respectively. Up to four post-edge features were observed, which have a U-*d* character with significant differences in terms of U-*d* and O-*p* cubic orbital mixtures. The pre-edge quadrupolar transition, U-2*p* → U-5*f*, previously observed in other uranium compounds is also reported in KUO_3_, but it is slightly overlapping with the crystal field split U-*d* *t*_2*g*_ line. At the uranium *M*_4_-edge, many identified spectral features show U–O bonding character as seen through the overlapping of U-*f* and O-*p* density of states, with significant differences between them when considering the calculated cubic harmonic of U-*f* and O-*p* orbitals. Moreover, the calculated degeneracy breaking of U-5*f* orbitals due to the octahedral crystal field and spin–orbit coupling is in line with the reported experimental forbidden Laporte *f*–*f* transition. Additional forbidden Laporte *f*–*f* transitions are also predicted, as well as potential charge transfer transitions and Laporte-allowed uranium 5*f* → 6*d* transitions, but they experimentally require optical spectra collected with very high resolution, which was not available when previous forbidden Laporte *f*–*f* transitions were reported. Calculated electron binding energies, reported between 0 and 400 eV, agree within a few eV with the only experimental data available, but also clearly indicate that new XPS analyses are mandatory to fully assess the electronic structure of KUO_3_. Finally, our results demonstrate that a combination of multi-edge HERFD-XAS can serve as a powerful tool to study the valence electronic structure of uranium, with the unique opportunity to assess in more detail the molecular orbitals.

## Supplementary Material

Supporting information file. DOI: 10.1107/S1600577521012431/yw5002sup1.pdf

## Figures and Tables

**Table 1:** Energy position of experimentally observed and calculated U *L*_3_-edge HERFD-XANES edge and post-edge spectral features; the U-*d* cubic harmonics mainly contributing to these features are also indicated

Feature	Experimental	Calculated	Involved U-*d* cubic harmonics
*t*_2*g*_	17173 (1) eV	17173.3 (2) eV	U-*d*_*xy*_, U-*d*_*xz*_, U-*d*_*yz*_
*e*_*g*_	17179.6 (5) eV	17180.2 (2) eV	U-d_{z^{2}}, U-d_{(x^{2}-y^{2})}
A	17191 (1) eV	17189.9 (2) eV	U-*d*_*xy*_, U-*d*_*xz*_, U-*d*_*yz*_
A{}^{\star}	17200 (1) eV	17197.1 (2) eV	U-*d*_*xy*_, U-*d*_*xz*_, U-*d*_*yz*_
B	17222.1 (5) eV	17219.5 (2) eV	U-d_{z^{2}}, U-d_{(x^{2}-y^{2})}
C	17238.7 (5) eV	17236.6 (2) eV	All

**Table 2:** Energy position of experimentally observed and calculated U *M*_4_-edge HERFD-XANES post-edge spectral features; the U-*f* cubic harmonics mainly contributing to these features are also indicated

Feature	Experimental	Calculated	Involved U-*f* cubic harmonics
A	3731.9 (5) eV	3732.1 (1) eV	U-f_{x^{3}}, U-f_{y^{3}}, U-f_{z^{3}}
B	3734.7 (5) eV	3734.7 (1) eV	U-*f*_*xyz*_
C	3738.3 (5) eV	3737.0 (1) eV	U-*f*_*xyz*_,
			U-f_{x^{3}}, U-f_{y^{3}}, U-f_{z^{3}}
D	3750.1 (5) eV	3746.5 (1) eV	U-*f*_*xyz*_,
			U-f_{x(y^{2}+z^{2})}, U-f_{y(x^{2}+z^{2})}, U-f_{z(x^{2}+y^{2})}
E	3757.3 (5) eV	3754.9 (1) eV	U-*f*_*xyz*_,
			U-f_{x(y^{2}+z^{2})}, U-f_{y(x^{2}+z^{2})}, U-f_{z(x^{2}+y^{2})}
F	3765.3 (5) eV	3763.1 (1) eV	U-f_{x^{3}}, U-f_{y^{3}}, U-f_{z^{3}}

**Table 3:** Crystal field splitting energies (all in eV) of the U-5*f* electronic levels above the Γ_7_ ground state in KUO_3_. Those peaks are contributing to the white line intensity in the U *M*_4_-edge HERFD-XANES spectra. Experimental forbidden Laporte *f*–*f* transition Γ_7_ → Γ_*n*_ from Kemmler-Sack (1968 ▸) and from Kanellakopulos *et al.* (1980 ▸) were initially reported in cm^−1^ units, and are here converted to eV units. Additional predicted transitions Γ_7_ → *X*_*n*_ are also indicated. The most significant U-*f* cubic harmonics for each state are also reported (see text for more details)

	This work	Kemmler-Sack		Kanellakopulos		
Transition	Calc.	Exp.	Calc.	Exp.	Calc.	Main U-*f* cubic harmonic involved
Γ_7_ → *X*_1_	0.133 (5)	–	–	–	–	U-*f*_*xyz*_
Γ_7_ → *X*_2_	0.179 (5)	–	–	–	–	U-f_{x(y^{2}+z^{2})},
						U-f_{y(x^{2}+z^{2})}, U-f_{z(x^{2}+y^{2})}
Γ_7_ → Γ_8_	0.501 (5)	0.543 (1)	0.551 (1)	0.564 (1)	0.554 (1)	U-f_{x(y^{2}+z^{2})},
						U-f_{y(x^{2}+z^{2})}, U-f_{z(x^{2}+y^{2})}
Γ_7_ → *X*_3_	0.645 (5)	–	–	0.678 (1)	0.676 (1)	U-*f*_*xyz*_, U-f_{x(y^{2}+z^{2})},
						U-f_{y(x^{2}+z^{2})}, U-f_{z(x^{2}+y^{2})}
Γ_7_ → \Gamma_{7}^{\star}	0.763 (5)	0.849 (1)	0.848 (1)	0.889 (1)	0.888 (1)	U-f_{x(y^{2}+z^{2})},
						U-f_{y(x^{2}+z^{2})}, U-f_{z(x^{2}+y^{2})}
Γ_7_ → *X*_4_	0.897 (5)	–	–	–	–	U-*f*_*xyz*_, U-f_{x(y^{2}+z^{2})},
						U-f_{y(x^{2}+z^{2})}, U-f_{z(x^{2}+y^{2})}
Γ_7_ → *X*_5_	1.025 (5)	–	–	–	–	U-f_{x^{3}}, U-f_{y^{3}},
						U-f_{z^{3}}, U-f_{x(y^{2}+z^{2})},
						U-f_{y(x^{2}+z^{2})}, U-f_{z(x^{2}+y^{2})}
Γ_7_ → \Gamma_{8}^{\star}	1.199 (5)	1.216 (1)	1.311 (1)	1.185 (1)	1.305 (1)	U-f_{x(y^{2}+z^{2})},
						U-f_{y(x^{2}+z^{2})}, U-f_{z(x^{2}+y^{2})}
Γ_7_ → *X*_6_	1.304 (5)	–	–	–	1.340 (1)	U-f_{x^{3}}, U-f_{y^{3}}, U-f_{z^{3}}
Γ_7_ → Γ_6_	1.582 (5)	1.549 (1)	1.589 (1)	1.550 (1)	1.558 (1)	U-f_{x^{3}}, U-f_{y^{3}}, U-f_{z^{3}}
Γ_7_ → *X*_7_	2.086 (5)	–	–	–	–	U-f_{x^{3}}, U-f_{y^{3}}, U-f_{z^{3}}
Γ_7_ → *X*_8_	2.352 (5)	–	–	–	–	U-f_{x^{3}}, U-f_{y^{3}}, U-f_{z^{3}}

**Table 4:** Electron binding energies (in eV) in KUO_3_ as deduced from the calculated density of states down to U-4*f* electrons. Energies given in brackets refer to the energy range where density of states show multiplet structure, which is especially the case in the valence molecular orbitals. Experimental data are from X-ray photoelectron spectroscopy experiments from Liu *et al.* (2009 ▸) and from Lopez *et al.* (2017 ▸). Experimental uncertainties in Liu’s data refer to the line width at half maximum

Atom-*nl*_*j*_	Calculated KUO_3_	XPS (Liu)	XPS (Lopez)
U-4*f*_5/2_	385.1 (1)	391.0 (1.9)	390.947
U-4*f*_7/2_	373.9 (1)	380.1 (1.8)	379.857
K-2*s*_1/2_	353.9 (1)	–	–
U-5*s*_1/2_	314.7 (1)	–	–
K-2*p*_1/2_	281.4 (1)	–	–
K-2*p*_3/2_	278.5 (1)	–	–
U-5*p*_1/2_	251.4 (1)	–	–
U-5*p*_3/2_	195.2 (1)	–	–
U-5*d*_3/2_	102.1 (1)	–	–
U-5*d*_5/2_	93.7 (1)	–	–
U-6*s*_1/2_	43.3 (1)	–	–
K-3*s*_1/2_	26.7 (1)	–	–
U-6*p*_1/2_ / O-2*s*	25.3 (2)	–	–
U-6*p*_3/2_ / O-2*s*	[12; 20]	–	–
K-3*p*_1/2_ / O-2*s* / O-2*p*	10.4 (3)	–	–
U-5*f*_5/2_ / K-3*p*_3/2_ / O-2*p*	[1; 6]	–	–
