# Supplementary material for: Application of multi-edge HERFD-XAS to assess the uranium valence electronic structure in potassium uranate (KUO3)
Source: J Synchrotron Radiat. 2022 Jan 1;29(Pt 1):21–9. doi: 10.1107/S1600577521012431 (PMC8733981; doi:10.1107/S1600577521012431)
Supplement: Supplementary file 1 [file s-29-00021-sup1.pdf]

JOURNAL OF SYNCHROTRON RADIATION

Volume 29 (2022)

Supporting information for article:

Application of multi-edge HERFD-XAS to assess the uranium valence electronic structure in potassium uranate (KUO3)

René Bes, Gregory Leinders and Kristina Kvashnina

Figure 6 shows the calculated density of states of KUO_3 for K, U and O atoms from 5 eV below the Fermi energy level (situated at 0) to 80 eV above it. s, p, d and f states are plotted separately for sake of clarity. This figure aims to support Figure 2, Figure 3 and Figure 4 by providing the density of states for other orbitals than the one directly involved in the corresponding XANES spectra interpretation.

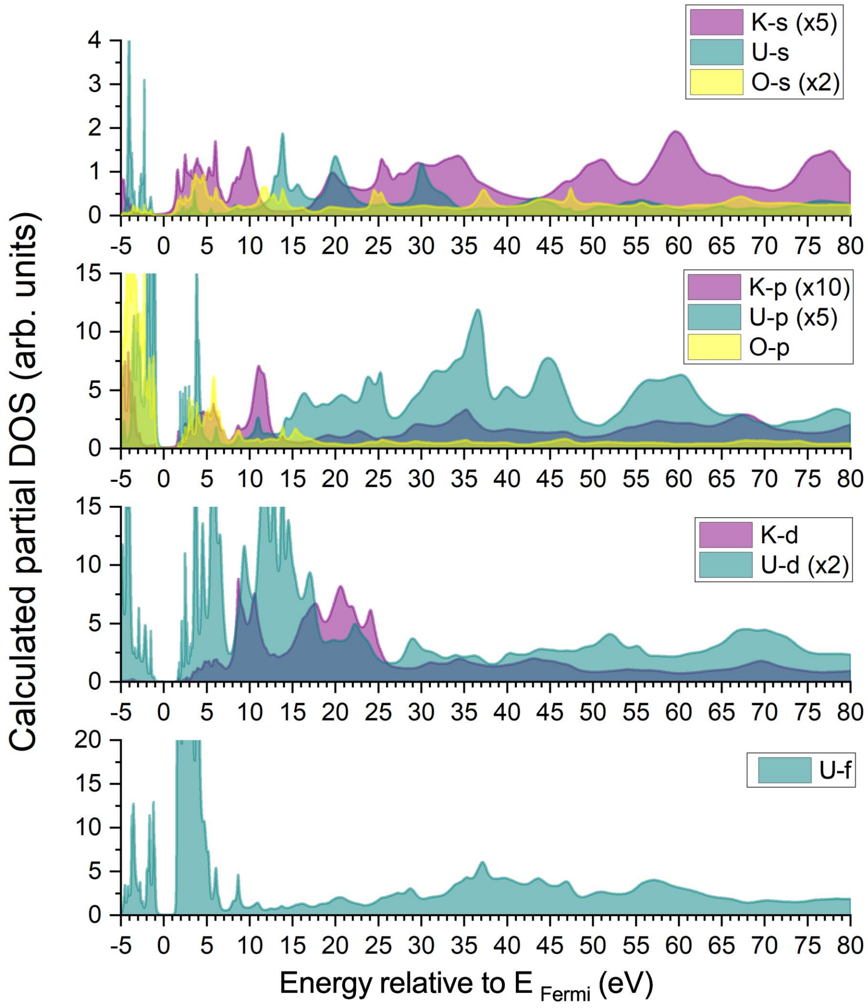

Fig. 6. Calculated density of states of KUO_3 for K, U and O atoms.

Figure 7 is the Uranium M_4-edge HERFD-XANES spectrum in full, complementing Figure 4 which is zoomed on the numerous satellite features above the whiteline. A zoom on the white line is also provided as an inset in order to show the slight asymmetry of the main peak, supporting discussions based on Figure 5.

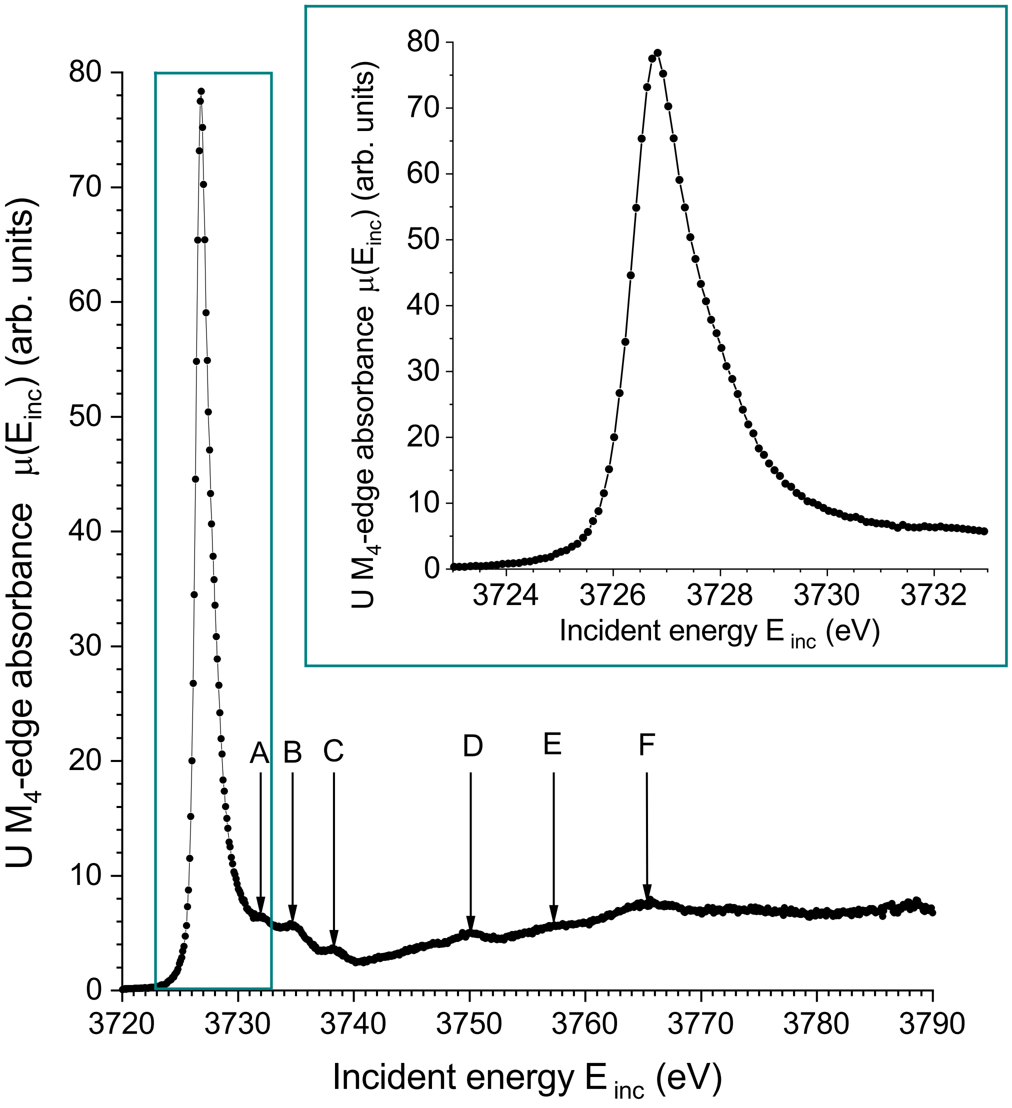

Fig. 7. Uranium M_4-edge HERFD-XANES spectrum. Inset is a zoom on the white line to show the slight asymmetry of the main peak.

Figure 8 is the X-ray diffractogram of the KUO_3 powder and Rietveld fit to the collected pattern. X-ray diffraction was performed using a PANalytical XPert Pro diffractometer, operating in a vertical Bragg-Brentano geometry. A Cu LFF X-ray tube was used as radiation source, and a Ni-filter was placed in the diffracted beam path to remove Cu K_β contribution. A positition-sensitive detector (PANalytical XCelerator) was used in scanning mode with an active opening of 2.1° (2θ). Soller slit assemblies (0.02 rad) were positioned in both incident and diffracted beam paths. The diffraction pattern was collected using a fixed incident divergence slit (1/2°). The sample was prepared by loading the polycrystalline KUO_3 powder into a back-loaded sample holder. Data treatment and Rietveld refinement was performed using Highscore Plus (v4.8).

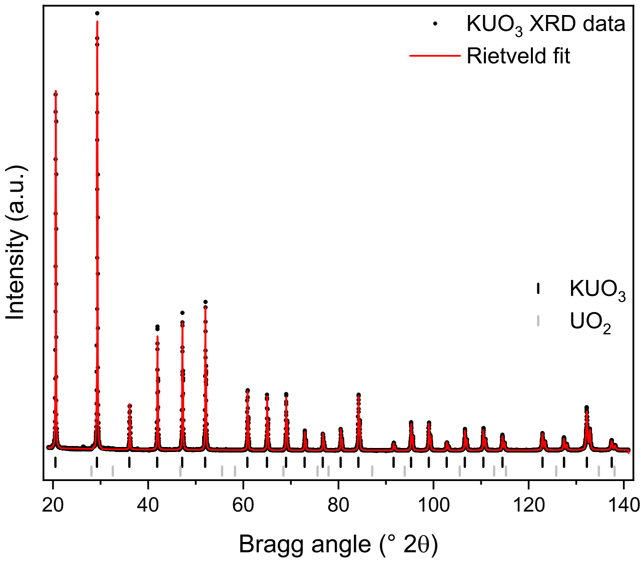

Fig. 8. X-ray diffractogram of the KUO_3 powder and Rietveld fit (Rwp = 7.4%) to the pattern. At the bottom of the graph, vertical lines indicate the position of reflections belonging to the KUO_3 phase (space group Pm$\bar{3}$m) and to the UO_2 phase (space group Fm$\bar{3}$m). The KUO_3 sample appears phase-pure and no remaining UO_2 phase could be detected.
